# Histopathological evaluation of dopamine receptor D2 expression in symptomatic gonadotroph pituitary neuroendocrine tumors: a case series including a rare metastatic case responsive to a dopamine agonist

**DOI:** 10.1007/s10014-025-00521-3

**Published:** 2025-11-16

**Authors:** Ken Uekawa, Naoki Shinojima, Takahiro Yamamoto, Shigeo Anai, Kozo Tashima, Motoyuki Igata, Takeshi Matsumura, Rumi Sasaki, Takashi Ohba, Hironori Tashiro, Kazuhito Tanaka, Kentaro Tanaka, Hiroyo Mabe, Shigetoshi Yano, Toshinori Hirai, Yoshiki Mikami, Akitake Mukasa

**Affiliations:** 1 Department of Neurosurgery, Kumamoto University Hospital, 1-1-1, Honjo, Chuo-ku, Kumamoto, 860-8556 Japan; 2 Department of Diabetes, Metabolism and Endocrinology, Kumamoto University Hospital, Kumamoto, 860-8556 Japan; 3 Department of Medical Technology, Faculty of Health Sciences, Kumamoto Health Science University, Kumamoto, 861-5598 Japan; 4 Department of Obstetrics and Gynecology, Kumamoto University Hospital, Kumamoto, 860-8556 Japan; 5 Department of Reproductive Health, Faculty of Life Sciences, Kumamoto University, Kumamoto, 862-0976 Japan; 6 Department of Diagnostic Pathology, Kumamoto University Hospital, Kumamoto, 860- 8556 Japan; 7 Department of Gastroenterology and Hepatology, Faculty of Life Sciences, Kumamoto University, Kumamoto, 860-8556 Japan; 8 Department of Pediatrics, Kumamoto University Hospital, Kumamoto, 860-8556 Japan; 9 Department of Neurosurgery, Minamifukuoka Neurosurgical Hospital, Fukuoka, 811-1313 Japan; 10 Department of Diagnostic Radiology, Faculty of Life Sciences, Kumamoto University, Kumamoto, 860-8556 Japan

**Keywords:** Functioning gonadotroph pituitary neuroendocrine tumors (Gn-PitNETs), Metastatic pituitary neuroendocrine tumors (PitNETs), Dopamine agonists, Dopamine receptor D2 (DRD2), Ovarian hyperstimulation

## Abstract

Functioning gonadotroph pituitary neuroendocrine tumors (Gn-PitNETs) cause ovarian hyperstimulation. We report Gn-PitNETs, including a distant metastasis case responsive to a dopamine agonist (DA), cabergoline. A 10-year-old girl presented with ovarian enlargement and elevated FSH levels (33.7 mIU/mL). A pituitary tumor was resected and confirmed as a Gn-PitNET. Radiotherapy for residual tumor improved ovarian enlargement. At age 20, ovarian enlargement recurred and cabergoline relieved symptoms and decreased FSH levels. At age 35, ovarian enlargement recurred after discontinuing cabergoline but improved with its reinstatement. Liver biopsy confirmed metastatic Gn-PitNET and DRD2 positivity. A 26-year-old woman presented with ovarian enlargement and elevated FSH levels (31.6 mIU/mL). A pituitary tumor was resected and confirmed as a Gn-PitNET with negative DRD2. FSH levels normalized, and ovarian size improved. A 48-year-old man presented with bitemporal hemianopia. FSH levels were elevated (251.4 mIU/mL) without testicular enlargement. The pituitary tumor was resected, improving hemianopia and normalizing FSH levels. Histopathology confirmed a Gn-PitNET with strong FSH staining and DRD2 positivity. In a rare case of metastatic Gn-PitNET, cabergoline was associated with tumor stability and hormone control. Further studies may clarify whether DRD2 expression can help predict DA responsiveness in refractory tumors.

(200/200 words)

**Supplementary Information　We have used “Supplementary” consistently in the manuscript and in the Supplementary Information files. If the journal style requires “Supplementary,” please adjust the wording throughout.:**

The online version contains supplementary material available at 10.1007/s10014-025-00521-3.

## Introduction

Pituitary neuroendocrine tumors (PitNETs) are classified as either functioning or non-functioning, depending on whether they cause hormone-related symptoms. Most non-functioning PitNETs are gonadotroph PitNETs (Gn-PitNETs), which express follicle-stimulating hormone (FSH) or luteinizing hormone (LH). However, functioning Gn-PitNETs are rare, comprising approximately 0.2% of all PitNETs [1].

Symptoms of functioning Gn-PitNETs depend on sex and hormone bioactivity. Premenopausal women typically present with oligomenorrhea, amenorrhea, infertility, galactorrhea, or ovarian enlargement, while men may develop testicular enlargement [2, 3]. The functional status of these tumors is influenced by FSH bioactivity, patient age, sex, and gonadal hormone sensitivity. Notably, even tumors with high hormone secretion may remain clinically non-functioning [2, 3].

Surgical resection remains the first-line treatment for Gn-PitNETs, whereas radiotherapy is reserved for refractory cases; however, long-term outcomes remain undefined. Medical therapies, including dopamine agonists (DAs) and somatostatin analogs, have shown limited success, with tumor shrinkage and symptom relief proving elusive [2, 3].

This case series presents two female patients with functioning Gn-PitNETs and ovarian enlargement, including a rare case with liver and bone metastases responding to DA therapy. Additionally, we describe a case of a male patient with silent Gn-PitNET with extremely high FSH levels and bilateral hemianopia, emphasizing the effect of surgical resection on FSH reduction and histopathological findings. Pathological analysis included dopamine receptor D2 (DRD2) expression, providing insights into the potential role of dopamine agonists in refractory or metastatic cases of functioning Gn-PitNETs.

## Clinical summary

### Case 1

At age 10, the patient presented with an acute abdomen and bitemporal hemianopia. Abdominal magnetic resonance imaging (MRI) revealed bilateral ovarian enlargement with polycystic follicles. Laboratory tests showed elevated FSH (33.7 mIU/mL), estradiol (E2, 3840 pg/mL), and low LH (< 0.5 mIU/mL). Head MRI identified a dumbbell-shaped tumor extending from the intrasellar to the suprasellar region. The tumor was resected via microscopic transsphenoidal surgery. Histopathology confirmed a PitNET with FSH and slight LH positivity (Supplementary Fig. 3). Owing to residual tumor and persistent hormonal elevation, 50 Gy local radiotherapy was performed, normalizing FSH and E2 levels, reducing ovarian size, and shrinking the tumor. These early management details were previously reported [4, 5]. She developed diabetes insipidus and hypopituitarism, requiring desmopressin, cortisol, thyroxine, growth hormone (GH), and estrogen-progesterone therapy. GH was paused at age 13, and resumed at age 18; the FSH levels gradually rose subsequently (Supplementary Fig. 1).

**Fig. 1:**
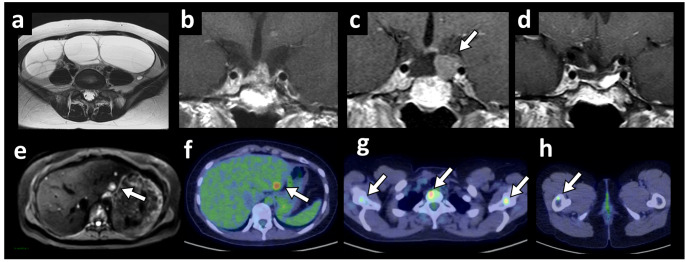
Radiographic findings of case 1. **a** Abdominal MRI T2-weighted image (T2WI) showing bilateral ovarian enlargement and polycystic follicles. **b** Head MRI T1-weighted image (T1WI) with gadolinium (T1-Gd) enhancement showing no remarkable recurrence of the pituitary tumor at age 20. **c** Head MRI T1-Gd showing tumor regrowth in the left cavernous sinus at age 28. **d** Head MRI T1-Gd showing no obvious tumor recurrence in the pituitary at age 35. **e** Abdominal MRI diffusion-weighted image showing multiple masses in the liver. **f**–**h** 18 F-fluorodeoxyglucose positron emission tomography (FDG-PET) scan showing metastases to the liver (**f**), multiple bone metastases in the cervical, thoracic, and lumbar spine, both scapulae (**g**), and the right femur (**h**)

**Fig. 2:**
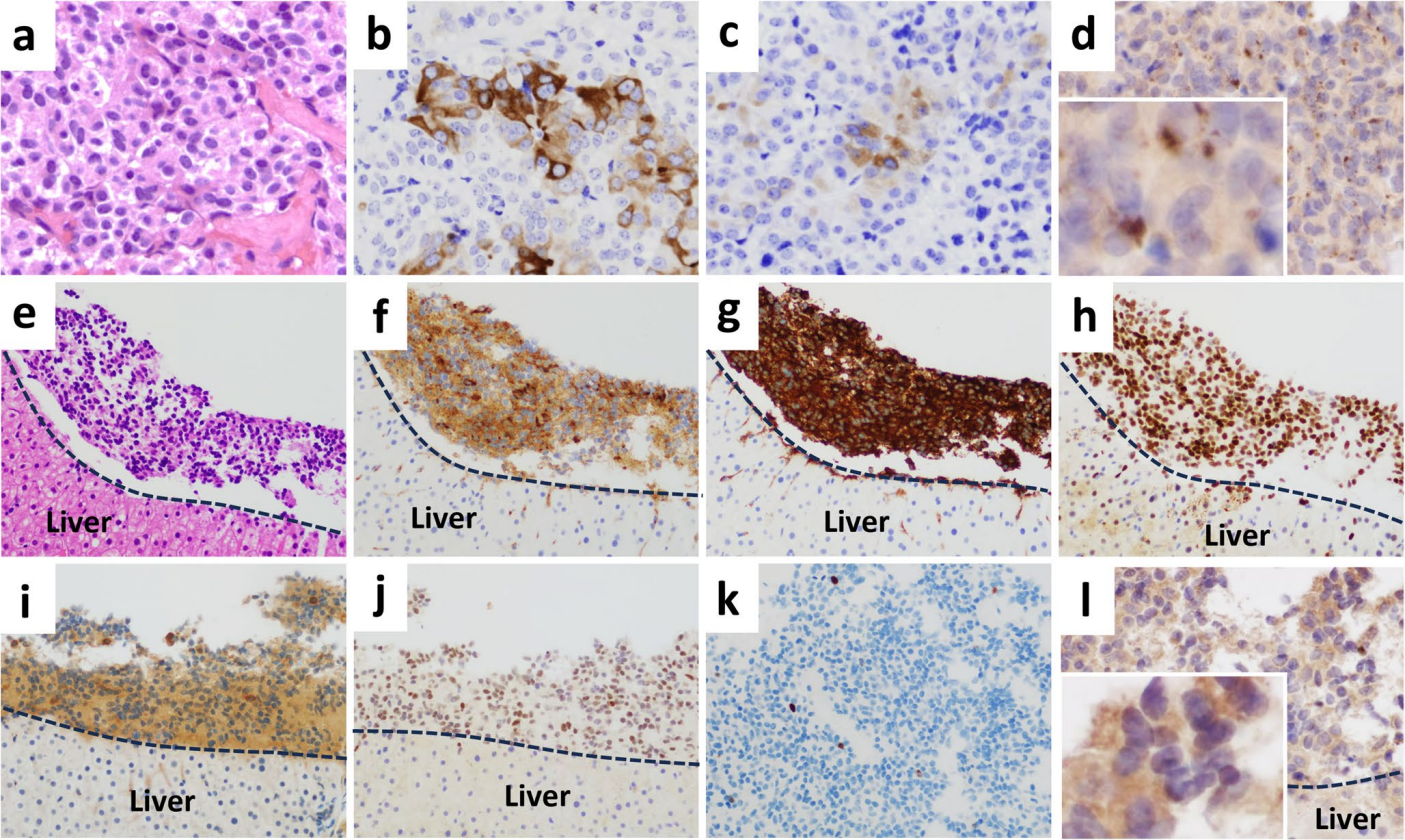
Pathological findings of case 1 . **a** Hematoxylin and eosin (HE) staining, **b** follicle-stimulating hormone (FSH), **c** luteinizing hormone (LH), and **d** dopamine receptor D2 (DRD2, cat. no. 324393, Calbiochem) from the pituitary tumor resection at age 20. **e** HE staining, **f** Chromogranin A, **g** Synaptophysin, **h** Insulinoma-associated protein 1 (INSM1), **i** FSH, **j** steroidogenic factor 1 (SF-1), **k** Ki-67, and **l** DRD2 from the liver biopsy at age 35

At age 20, ovarian enlargement and hormonal elevation recurred without MRI evidence of tumor (Fig. 1a, b). Cavernous sinus sampling showed no central-peripheral FSH gradient (peripheral: 9.6–10.3 mIU/mL; right and left cavernous sinuses: 9.4–11.1 mIU/mL). Bromocriptine, a dopamine agonist, partially suppressed FSH; octreotide was ineffective (Supplementary Fig. 2a-b). A second endoscopic transnasal resection confirmed recurrent PitNET with FSH and slight LH positivity (Fig. 2a-c and Supplementary Fig. 3). Ki-67 was < 1% and, DRD2 staining was positive (Fig. 2d). Cabergoline (0.25 mg/week), another dopamine agonist, reduced FSH and ovarian size. Despite therapy, tumor recurred at age 28 (Fig. 1c) without FSH elevation or ovarian enlargement. Resection revealed FSH-negative pathology (Supplementary Fig. 3); cabergoline maintained disease stability.

At age 35, during infertility treatment, cabergoline was stopped, leading to ovarian enlargement; FSH 21.7 mIU/mL and E2 1710 pg/mL were initiated. Head MRI showed no recurrence (Fig. 1d), but abdominal MRI found multiple liver masses (Fig. 1e), and 18 F-fluorodeoxyglucose positron emission tomography (FDG-PET) revealed metastases in the liver, bones, and cavernous sinus (Fig. 1f-h). Indium-111 pentetreotide scintigraphy (OctreoScan^®^) a nuclear imaging test for detecting somatostatin receptor-positive neuroendocrine tumors, was negative. Liver biopsy showed PitNET-like cells (Fig. 2e) with positive for neuroendocrine markers (Fig. 2f-h) and for FSH (Fig. 2i), with Ki-67 < 1% (Fig. 2k). DRD2 positivity (Fig. 2l) supported cabergoline use. As in the primary PitNET, steroidogenic factor 1 (SF-1/NR5A1) was positive (Fig. 2j), while pituitary-specific positive transcription factor 1 (PIT1/POU1F1) and T-box pituitary transcription factor (TPIT/TBX19) were negative (Supplementary Fig. 4), supporting gonadotroph lineage differentiation. Cabergoline (0.5 mg daily for 10 days) was administered based on ovarian hyperstimulation syndrome (OHSS) treatment protocols, followed by 0.25 mg once weekly as maintenance, sustaining FSH reduction and improvement in ovarian enlargement. Though not ideal during fertility treatment, cabergoline controlled tumor activity. Over three years, MRI and FDG-PET showed stable disease and tumor shrinkage.

### Case 2

**Fig. 3:**
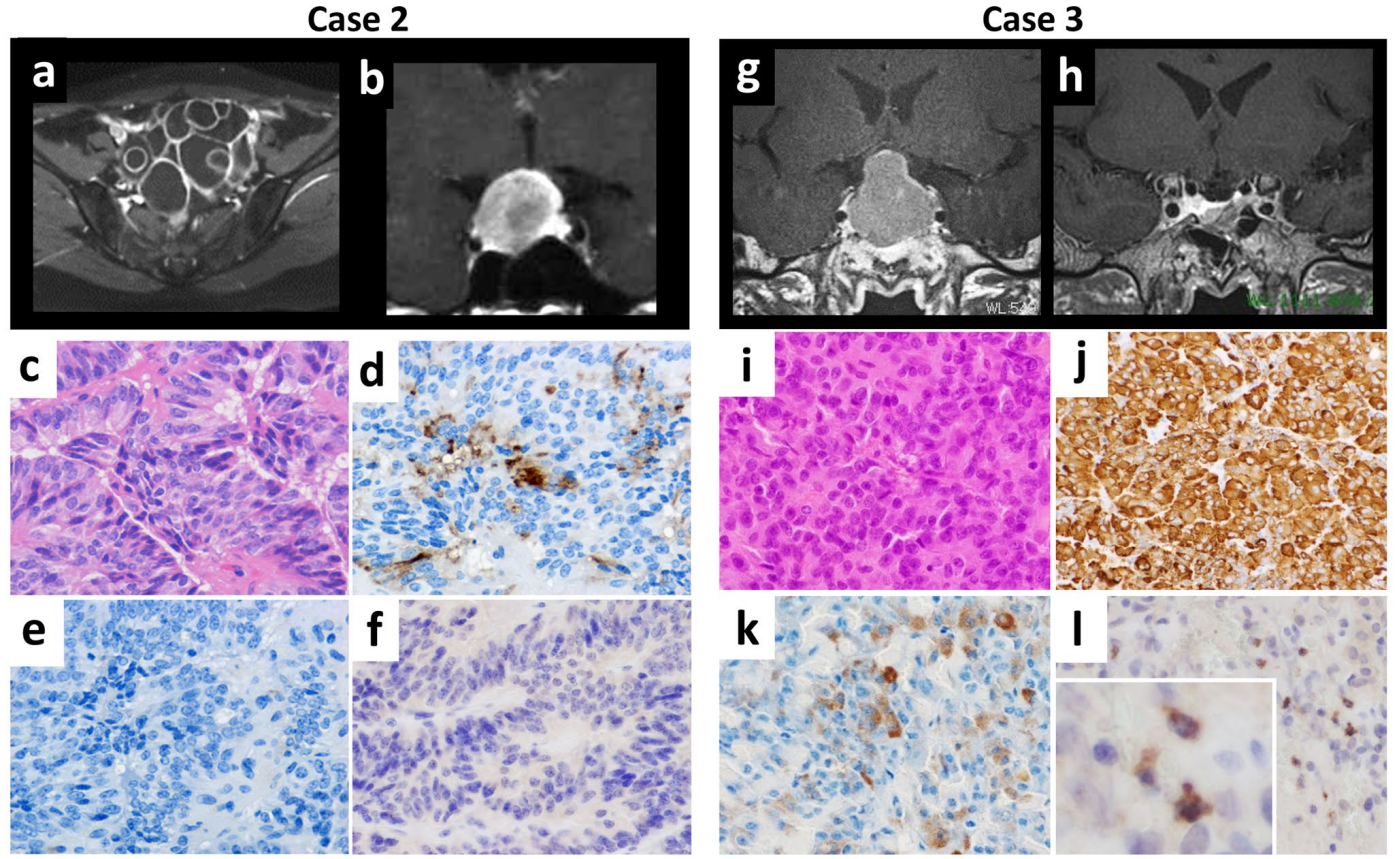
Magnetic resonance imaging (MRI) and pathological findings of case 2 (**a**–**f**) and case 3 (**g**–**l**). **a** Abdominal T1-weighted image (T1WI) with gadolinium enhancement (T1-Gd) showing bilateral ovarian enlargement and polycystic follicles. **b** Head MRI T1-Gd showing a pituitary tumor. **c** Hematoxylin and Eosin (HE) staining, and immunostaining for **d** follicle-stimulating hormone (FSH), **e** luteinizing hormone (LH), and **f** dopamine receptor D2 (DRD2, cat. no. 324393, Calbiochem) in case 2. **g** Head MRI T1-Gd showing a pituitary tumor. **h** Post-treatment head MRI T1-Gd showing the resected pituitary tumor. **i** HE staining, and immunostaining for **j** FSH, **k** LH, and **l** DRD2

A 26-year-old woman presented with abdominal pain and bitemporal hemianopia. Abdominal MRI revealed bilateral ovarian enlargement (Fig. 3a); tests revealed FSH 31.6 mIU/mL, E2 707.9 pg/mL, LH < 0.2 mIU/mL. Head MRI revealed a pituitary tumor (Fig. 3b). Endoscopic resection normalized hormone levels (FSH < 1.0 mIU/mL, E2 < 25.0 pg/mL), and ovarian size improved and reduced ovarian size. No residual tumor was seen on follow-up MRI. Histopathology confirmed Gn-PitNET with partial FSH positivity, negative LH (Fig. 3c-e), Ki-67 < 1%, and negative DRD2 (Fig. 3f). Ovarian enlargement has not recurred in the following 20 years.

### Case 3

A 48-year-old man presented with bitemporal hemianopia; MRI showed a tumor compressing the optic nerve (Fig. 3g). FSH was 251.4 mIU/mL, LH 2.4 mIU/mL, and testosterone 414.9 ng/dL. No testicular enlargement was noted. Bromocriptine partially suppressed FSH (Supplementary Fig. 2c). Endoscopic surgery resolved visual symptoms and normalized FSH (7.2 mIU/mL). Histopathology showed Gn-PitNET with strong FSH and partial LH positivity (Fig. 3i-k), Ki-67 < 1%, and partial DRD2 positivity (Fig. 3l). Testosterone levels later declined, requiring replacement therapy.

### Pathological findings

#### Immunohistochemistry

Formalin-fixed, paraffin-embedded sections were stained using following antibodies with validated controls: FSH (Clone C10, Dako, CA, USA) at 1:100, LH (Clone C93, Dako, CA, USA) at 1:100, Chromogranin A (clone 5H7, NCL-CHROM-430, Leica Biosystems, Newcastle upon Tyne, UK) at 1:400, Synaptophysin (clone 27G12, Leica Biosystems, Newcastle upon Tyne, UK) undiluted, Insulinoma-associated protein 1 (INSM1)(clone A-8, Santa Cruz Biotechnology, Santa Cruz, CA, USA) at 1:200, SF-1 (clone N1665, Perseus Proteomics, Tokyo, Japan) at 1:200, PIT1 (cat. no. ab235915, Abcam, Cambridge, UK) at 1:500, TPIT (cat. no. MA5-31431, Thermo Fisher Scientific, Waltham, MA, USA) at 1:1000, somatostatin receptor (SSTR): SSTR2A (clone EP149, Nichirei, Tokyo, Japan) at 1:4, and SSTR5 (AB5681, Chemicon, Temecula, CA, USA) at 1:800, prolactin (cat. no. A0569, DAKO, Glostrup, Denmark) at 1:500, growth hormone (GH) (cat. no. N1561, DAKO, Glostrup, Denmark) undiluted, thyroid-stimulating hormone (TSH) (cat. no. M3503, DAKO, Glostrup, Denmark) at 1:100, and adrenocorticotropic hormone (ACTH) (cat. no. M3501, DAKO, Glostrup, Denmark) at 1:100. DRD2 immunostaining was performed with two rabbit polyclonal antibodies: Calbiochem (cat. no. 324393; Darmstadt, Germany) at 1:500, and Bioss (cat. no. bs-1008R; Woburn, MA, USA) at 1:200. Human hippocampal tissue obtained at selective amygdalohippocampectomy for hippocampal sclerosis–related temporal lobe epilepsy, known to harbor D2-positive cells, was used as the positive control (Supplementary Fig. 5). Immunohistochemical specimens for DRD2, SSTR2A, and SSTR5 were evaluated by a single pathologist.

#### Histomorphological features

Histopathologic diagnosis was rendered according to the WHO Classification of Tumours, 5th edition (2022), *Endocrine and Neuroendocrine Tumours*, “Pituitary Tumours” section.

**Case 1**: Serial comparison of pituitary tumor resections at ages 10, 20, and 28 (Fig. 2a–d and Supplementary Fig. 3) showed, on HE sections, a densely cellular neoplasm composed of small round-to-oval nuclei, consistent with a PitNET. No significant cytologic atypia, tumor necrosis, or increased mitotic activity was identified at any time point. The Ki-67 labeling index was < 1% at age 20 and rose slightly to 2–3% at age 28. IHC demonstrated FSH positivity and weak/focal LH positivity at ages 10 and 20, whereas both FSH and LH were negative at age 28. SF-1 was positive, whereas PIT1 and TPIT were negative, consistent with gonadotroph lineage (Supplementary Fig. 4). Across all time points, tumor tissue was weakly positive for prolactin, whereas GH, TSH, and ACTH were negative (data not shown).

Liver biopsy (Fig. 2e–l): HE showed a dense proliferation of small round tumor cells within hepatic parenchyma, morphologically resembling the sellar tumor and supporting metastatic PitNET. Neuroendocrine markers (chromogranin A, synaptophysin, INSM1) were positive. The Ki-67 index was < 1%. As previously described, lineage markers were FSH positive and SF-1 positive, whereas PIT1 and TPIT were negative (Fig. 2 and Supplementary Fig. 4).

**Case 2**: Pituitary tumor (Fig. 3c–f): HE revealed papillary growth of tumor cells with round nuclei; in areas, tall columnar cells formed perivascular rosettes. No malignant features were identified. The Ki-67 index was < 1% (data not shown). IHC showed FSH positivity (Fig. 3d) with weak/focal LH positivity (Fig. 3e). SF-1 was positive, whereas PIT1 and TPIT were negative (Supplementary Fig. 4).

**Case 3**: Pituitary tumor (Fig. 3i–l): HE confirmed a PitNET without marked atypia, necrosis, or increased mitotic activity. IHC demonstrated FSH positivity (Fig. 3j) and LH positivity in ~ 10–20% of tumor cells (focal) (Fig. 3k). The Ki-67 labeling index was < 1% (data not shown). SF-1 was positive, whereas PIT1 and TPIT were negative (Supplementary Fig. 4).

#### Evaluation of DRD2 expression

As cabergoline was effective in Case 1, DRD2 immunostaining was exploratorily performed on both the primary pituitary and metastatic liver tumors to assess the potential mechanism of drug response. To investigate expression patterns, DRD2 staining was also applied to the other cases.DRD2 expression was evaluated using the Histo-score (H-score) [6, 7], Wang’s [8], and Vieira’s score [9]. The H-score combines staining intensity (0–3+) and percentage of positive cells, with scores ≥ 50 considered positive. Wang’s score evaluates intensity (0–3) and extent (0–4), with total scores > 2 considered as positive (2–3: low, > 3: high expression). Vieira’s score categorizes the percentage of stained cells as 0 (< 25%), 1 (25–50%), and 2 (> 50%). Nuclear staining was evaluated using the Allred score [10], combining proportion (0–5) and intensity (0–3) for a total score of 0–8. Scores 0–2 were negative and 3–8 were positive. Membranous staining was evaluated using the HER2 [7, 11] and Volante’s scores [12]. The HER2 score was originally developed for evaluating human epidermal growth factor receptor-2 (HER2) expression in breast carcinoma. It applies a four-point scale (0, 1+, 2+, 3+) based on the intensity, completeness, and extent of membranous staining. A score of 0 indicates no staining or membranous staining in $\:<$10% of tumor cells; 1+ indicates weak or incomplete membranous staining in $\:>$10% of cells; 2+ indicates moderate complete staining; and 3+ indicates strong complete membranous staining. Tumors with a HER2 score of 2+ or 3+ were considered positive. Volante’s score assesses SSTR2A expression in pituitary and neuroendocrine tumors based on subcellular localization and extent of staining. It uses a four-point scale (0, 1, 2, 3). A score of 0 indicates no staining; 1 indicates cytoplasmic staining only; 2 indicates membranous staining in <50% of tumor cells; and 3 indicates circumferential membranous staining in >50% of tumor cells. For analysis, scores of 2–3 were considered positive and 0–1 negative.

**Fig. 4:**
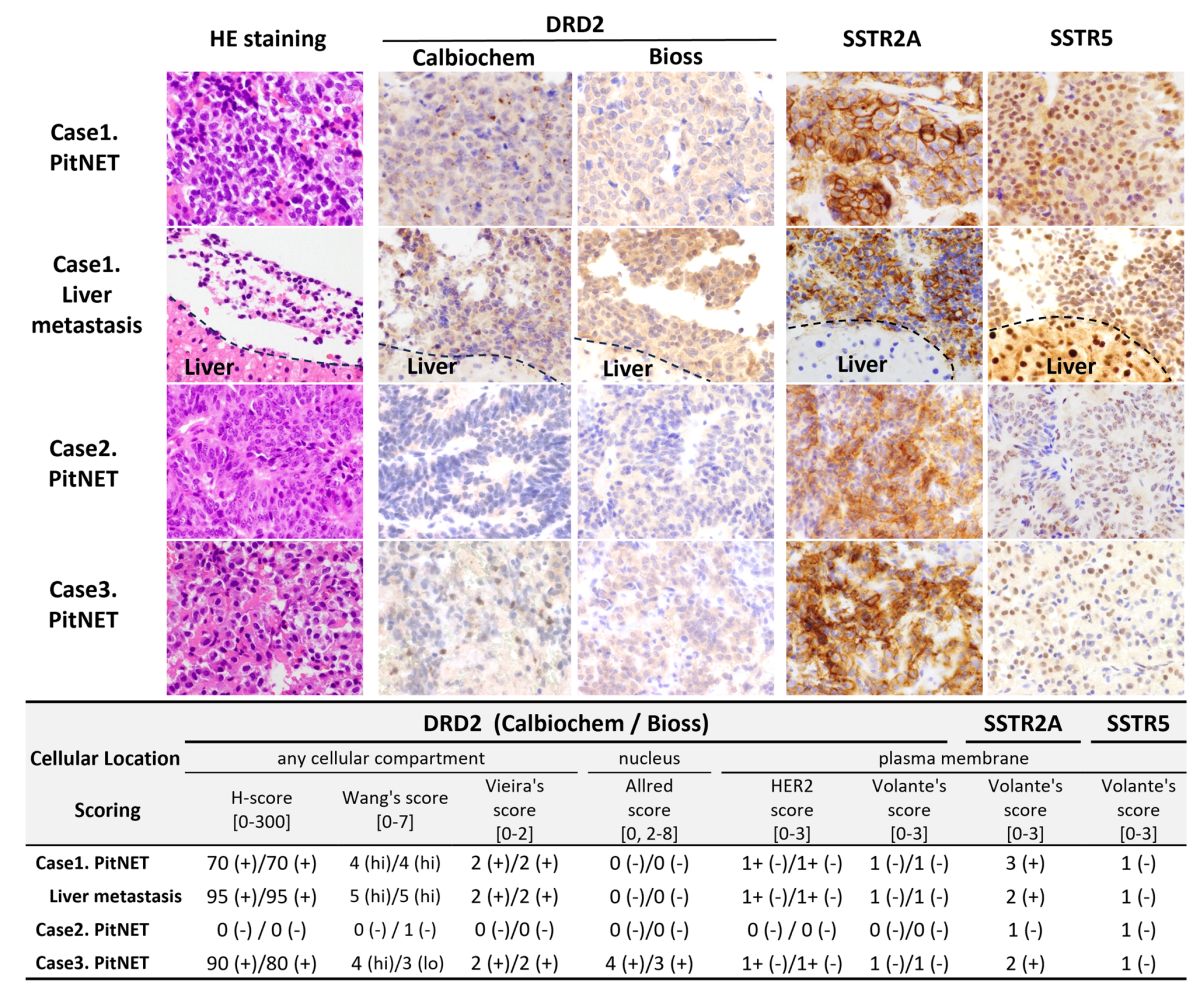
Pathological analysis in case 1–3 . Hematoxylin and Eosin (HE) staining, and immunostaining for dopamine receptor D2 (DRD2; cat. no. 324393, Calbiochem and cat. no. bs-1008R, Bioss) and somatostatin receptors (SSTR): SSTR2A and SSTR5. In the table below, multiple scoring methods are used to assess various staining properties for each marker. The values in brackets indicate the score range. The values in parentheses indicate the corresponding interpretation (negative, positive or low/high expression). PitNET: pituitary neuroendocrine tumor

Because DRD2 immunoreactivity could be antibody-dependent and some antibodies can yield non-specific or misleading patterns (e.g., apparent membranous or nuclear labeling), as previously described, we used two DRD2 (Calbiochem, cat. no. 324393; Bioss, cat. no. bs-1008R) to ensure an objective and reliable assessment, and added the comparative results in Fig. 4. In the results, for DRD2, cytoplasmic staining was predominantly observed in the PitNET and liver metastasis tissues of case 1, and in the PitNET tissue of case 3, whereas staining was minimal in the PitNET tissue of case 2. In case 3, partial nuclear staining of DRD2 was noted. Membranous staining for DRD2 was generally weak across all cases (Fig. 4). The Calbiochem antibody occasionally showed stronger cytoplasmic and focal nuclear labeling, with granular, intensely staining foci; however, overall cytoplasmic staining patterns were largely concordant between the two antibodies in samples from Cases 1–3 (Fig. 4) and in human hippocampus as positive control (Supplementary Fig. 5). Given the inherent limitations of DRD2 IHC, among the multiple scoring systems used in this study, the H-score most closely reflected the magnitude of cytoplasmic staining across cases. SSTR2A and SSTR5 expression were evaluated based on Volante’s score, and SSTR2A showed membranous staining in the PitNET and liver metastasis tissues of case 1 and in the PitNET tissue of case 3, but staining was poor in the PitNET tissue of case 2. SSTR5 exhibited only minimal membranous staining in all cases. The detailed scores for each case are summarized in the table within Fig. 4.

## Discussion

This case series describes two female patients with functioning Gn-PitNETs presenting with ovarian enlargement and one male patient with a silent Gn-PitNET characterized by markedly elevated FSH and bitemporal hemianopia. Notably, one female case (case 1) involved an aggressive, rare metastatic tumor with liver and bone involvement that responded to DA therapy. In Case 1, the hepatic metastasis was DRD2-positive, and DA therapy led to shrinkage of the metastatic lesions in the liver and bone with normalization of FSH levels.

Historically, aggressive pituitary tumors with malignant potential were referred to as “pituitary carcinoma.” However, the WHO 2022 Classification of Pituitary Tumors redefined PitNETs with metastatic behavior as “metastatic PitNETs”, acknowledging their poor responses to multimodal therapies [13–15]. Currently, there is no established grading or staging system for PitNETs, which remains an important area for future research. One recognized marker of malignancy is the Ki-67 index, where tumors with Ki-67 > 3% often exhibit malignant potential, and those with Ki-67 > 10% are considered highly aggressive [14–17]. Regarding metastatic behavior, metastases to the brain, bones (e.g., ilium), liver, and lungs have been reported, with ACTH-producing corticotroph PitNETs being the most common histopathological subtype, though non-functioning silent Gn-PitNETs have also been documented [14, 16, 17]. To our knowledge, there have been no previous reports of a functioning Gn-PitNET presenting with liver and multiple bone metastases, as observed in case 1, despite a low Ki-67 index of approximately 1%. Differences in metastatic behavior among PitNET subtypes may be attributed to variations in tumor aggressiveness, invasiveness, and the hormonal microenvironment.

In case 1, FDG-PET proved useful for whole-body screening. Despite immunohistochemical positivity for SSTR2A and SSTR5, OctreoScan^®^, a nuclear medicine imaging technique that detects somatostatin receptor expression in neuroendocrine tumors, failed to detect the metastatic lesions. The differential diagnosis should also include ectopic gonadotropin-producing tumors, such as mediastinal primaries [18]. As observed in case 1, although the Ki-67 index was low, indicating limited proliferative potential, long-term follow-up is essential given the risk of delayed distant metastasis. Furthermore, there is a need to elucidate the mechanisms of metastasis and to refine classification and treatment strategies.

Most Gn-PitNETs are non-functioning, and functioning Gn-PitNETs are rare, with variable clinical presentations, although their treatment strategies remain undefined. According to Ntali et al., surgical resection is the primary treatment for these tumors [2, 3]. In our cases, complete resection led to FSH normalization in cases 2 and 3, with case 2 also achieving sustained improvement in ovarian enlargement without recurrence. However, the optimal management of residual tumors in refractory cases remains a challenge. In case 1, a large suprasellar tumor was identified at age 10. Partial resection followed by radiotherapy initially resulted in FSH reduction and improvement in ovarian enlargement. However, recurrence at age 20 led to insufficient FSH reduction despite additional surgery. DA therapy, particularly cabergoline, was effective in this case. Ntali et al. reported that the effectiveness of DA and somatostatin analogs is limited, and no predictive markers for treatment response have been established. Therefore, therapeutic trials should be considered in individual cases [2, 3].

Several case reports suggest that certain functioning Gn-PitNETs exhibit exceptional sensitivity to DA therapy [2, 3]. A recent systematic review found that DA treatment led to a mild reduction in tumor secretion or ovarian size in 8 out of 18 cases [19]. In case 1, a DA administration test showed partial effectiveness, prompting the use of cabergoline, which resulted in a clinically significant response. DA therapy has also been reported to be effective for OHSS [2, 3]. Given this, in cases presenting with ovarian enlargement, initial DA therapy may be beneficial, and continued administration could be explored for potential tumor-suppressive effects.

Recent studies have clarified the mechanisms of the DA cabergoline action in lactotroph PitNETs. Cabergoline inhibits PRL secretion and tumor growth through multiple pathways, including protein synthesis inhibition, proliferation suppression, apoptosis induction, and fibrosis promotion, influencing the tumor microenvironment [20]. Additionally, recent studies have explored the multiple mechanisms of DA’s action in Cushing’s syndrome [21]. Despite widespread DRD2 expression in PitNETs, only a subset of cases respond to DA therapy, while others exhibit resistance [22]. The mechanisms underlying DA resistance remain unclear. Interestingly, a recent study reported that DRD2 expression based on 18 F-fallypride PET/MR predicts DA resistance in prolactinomas [23]. Loss of DRD2 function or alterations in intracellular signaling pathways may contribute to DA resistance in certain cases. Further research is needed to establish predictors of DA sensitivity.

In our study, immunohistochemical analysis of DRD2 expression was performed using multiple scoring methods. Positive staining was observed in 2 of 3 cases, and notably, DRD2 positivity was maintained in the liver biopsy specimen of the metastatic lesion in case 1. The staining pattern was predominantly cytoplasmic, as assessed by the H-, Wang’s, and Vieira’s scores, indicating that evaluation methods focusing on cytoplasmic immunoreactivity are more suitable for assessing DRD2 expression. Contrastingly, scoring systems based on membranous (Volante’s and HER2 score) or nuclear staining (Allred score) appeared to be less applicable. Previous studies have evaluated DRD2 expression in PitNETs. Wang et al. reported that high DRD2 expression (Wang’s score > 3) was observed in 128 of 197 PitNET cases (65%), including 31 of 37 Gn-PitNET cases (84%) [8]. Additionally, studies on non-functioning PitNETs demonstrated DRD2 expression in all tumors evaluated by immunohistochemistry and real-time RT-PCR, with most cases exhibiting tumor shrinkage under cabergoline therapy [9]. To our knowledge, no previous studies have evaluated DRD2 expression in tissue samples using multiple immunohistochemical scoring systems, and thus, the present analysis provides valuable insights. Further investigation is needed to clarify the relationship between DRD2 immunoreactivity and clinical outcomes or the efficacy of DA therapy. Because quantitative IHC assessment of DRD2 remains challenging and antibody-dependent, we corroborated our findings with two antibodies (Calbiochem, cat. no. 324393, and Bioss, cat. no. bs-1008R) and added the comparative results in Fig. 4. The Calbiochem antibody occasionally yielded stronger cytoplasmic and focal nuclear labeling, but overall cytoplasmic staining patterns were largely concordant between antibodies. Although DRD2 IHC has methodological limitations, in our series the H-score appeared to better reflect cytoplasmic staining than other systems. As with SSTR2A, for which optimized antibodies improved scoring [24], further refinement of DRD2 antibodies is expected to enhance reproducibility and quantification. Although evaluating DRD2 function and predicting response to DAs are difficult, a therapeutic trial of a DA is warranted in aggressive/metastatic PitNETs with limited treatment options.

When considering pharmacological treatments other than DAs, somatostatin analogs represent another therapeutic option. Gn-PitNETs show subtype differences, including SSTR expression, that might have implications for therapy [25]. In case 1, somatostatin analogs had limited efficacy in drug administration tests. However, given the presence of SSTR2A and SSTR5 expression, somatostatin analogs could still be considered as future treatment options. Furthermore, while no established grading system exists for PitNET malignancy [13, 15], as with other PitNET subtypes, temozolomide (TMZ) may be a treatment option if the tumor progresses to a high-grade state and becomes resistant to DAs. However, TMZ response is limited, and some tumors develop secondary resistance over time [26]. In case 1, TMZ was considered inappropriate owing to low Ki-67 and ongoing infertility treatment, where fetal safety was a concern. Additionally, the efficacy of antibody therapies, including anti-VEGF agents such as bevacizumab, remains unclear in refractory cases, warranting further investigation [8, 22].Our findings highlight the potential utility of immunohistochemical DRD2 assessment in guiding DA therapy for Gn-PitNETs. Standardized evaluation methods and further molecular studies are needed to support personalized treatment strategies in these rare but clinically significant tumors.

## Supplementary Information

Below is the link to the electronic supplementary material.

Supplementary Material 1

## Data Availability

The data that support the findings of this study are available from the corresponding author, Ken Uekawa, upon reasonable request.
