# Supplementary material for: Histopathological evaluation of dopamine receptor D2 expression in symptomatic gonadotroph pituitary neuroendocrine tumors: a case series including a rare metastatic case responsive to a dopamine agonist
Source: Brain Tumor Pathol. 2025 Nov 16;43(3):123–31. doi: 10.1007/s10014-025-00521-3 (PMC13375684; doi:10.1007/s10014-025-00521-3)
Supplement: Supplementary file 1 — Supplementary Material 1 [file 10014_2025_521_MOESM1_ESM.docx]

**<Supplemental Figure>**


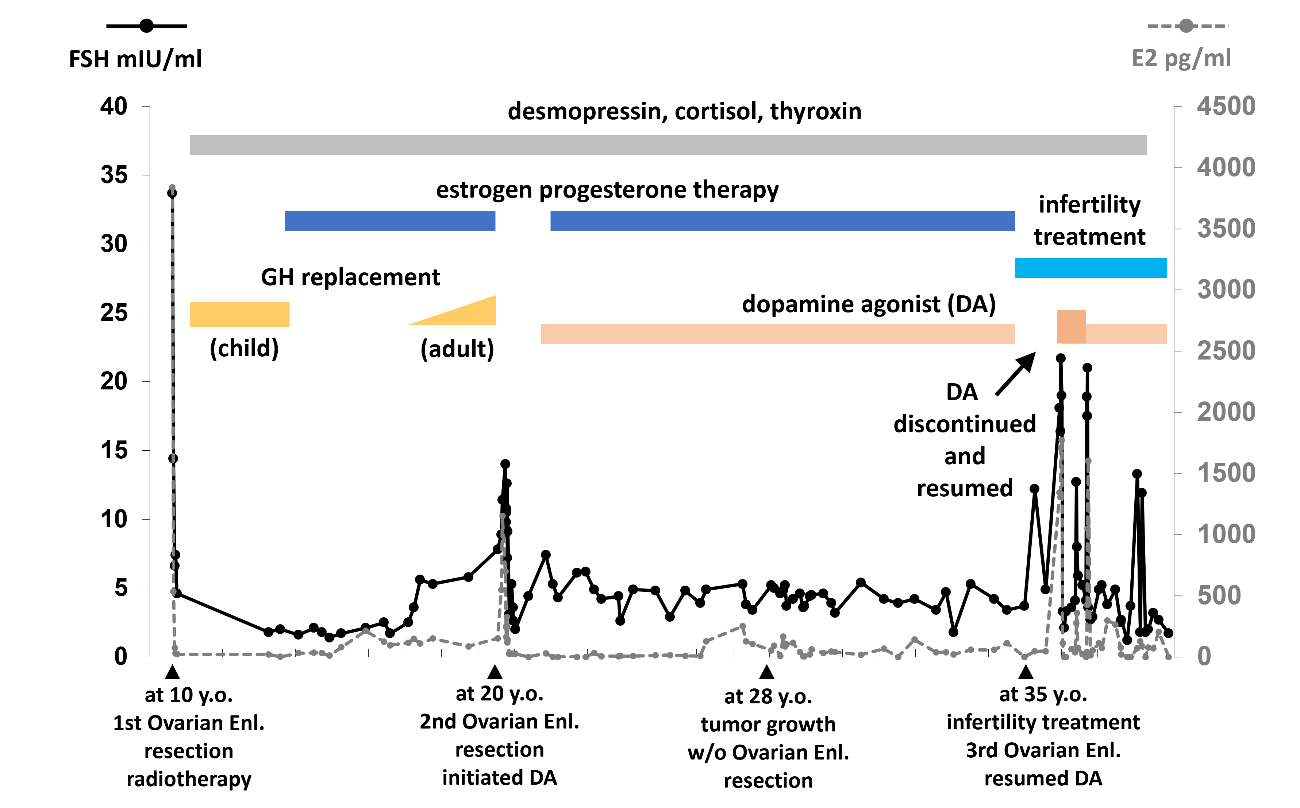


**Supplemental Fig. 1: Treatment course and hormone level trends in case 1**

The graph illustrates the clinical course with follicle-stimulating hormone (FSH) levels shown as a solid black line and E2 levels as a dotted gray line. The left axis represents FSH levels, while the right axis represents E2 levels. GH: growth hormone, DA: dopamine agonist, Ovarian Enl.: ovarian enlargement.


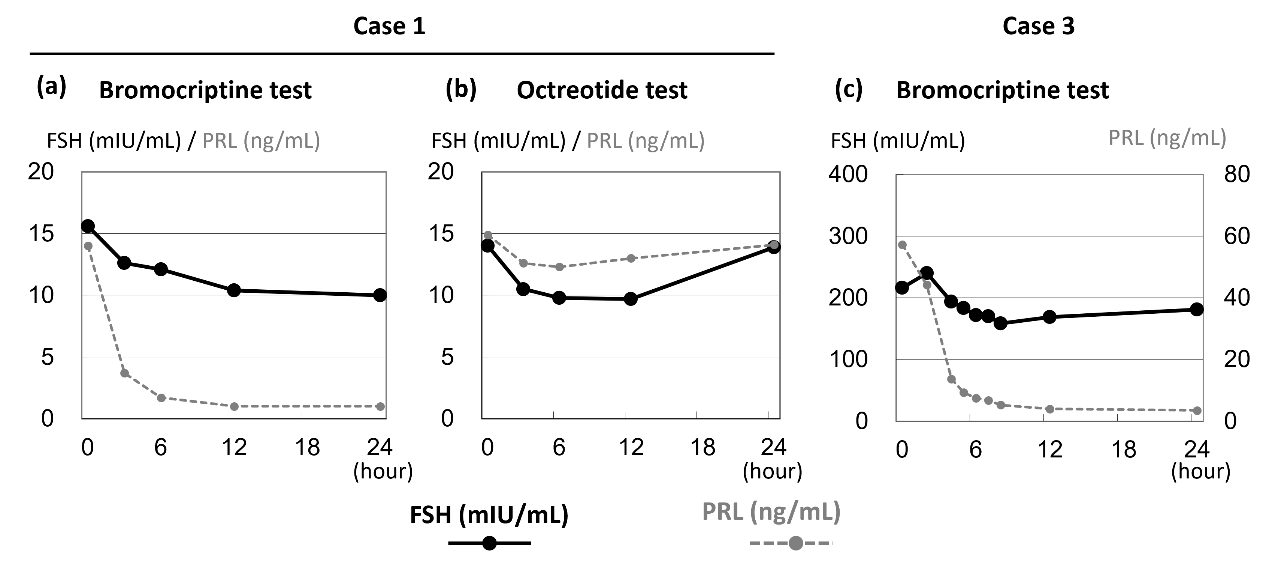


**Supplemental Fig. 2: Assessment of drug treatment response in case 1 (a-b) and case 3 (c)**

Administration test of (a) dopamine agonist, bromocriptine and (b) somatostatin analogue, octreotide in case 1, and (c) bromocriptine in case 3. FSH: follicle-stimulating hormone, PRL: prolactin.


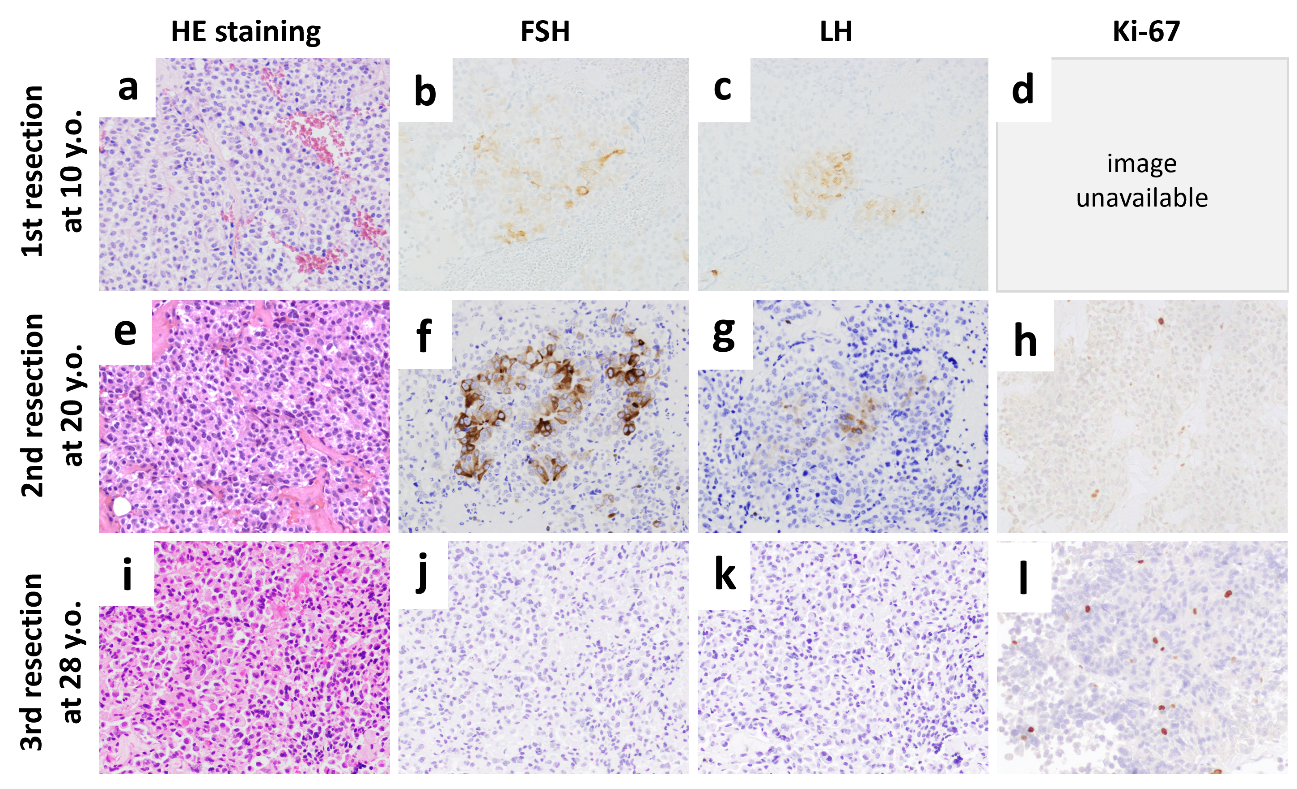


**Supplemental Figure 3: Serial pathological comparison in Case 1**

Rows correspond to resections at age 10 (a–d), age 20 (e–h), and age 28 (i–l). Columns show Hematoxylin–eosin (HE) (a, e, i), follicle-stimulating hormone (FSH) (b, f, j), luteinizing hormone (LH) (c, g, k), and Ki-67 (h, l). Panel (d) is not shown (image unavailable due to exhausted specimen). Hematoxylin counterstain.


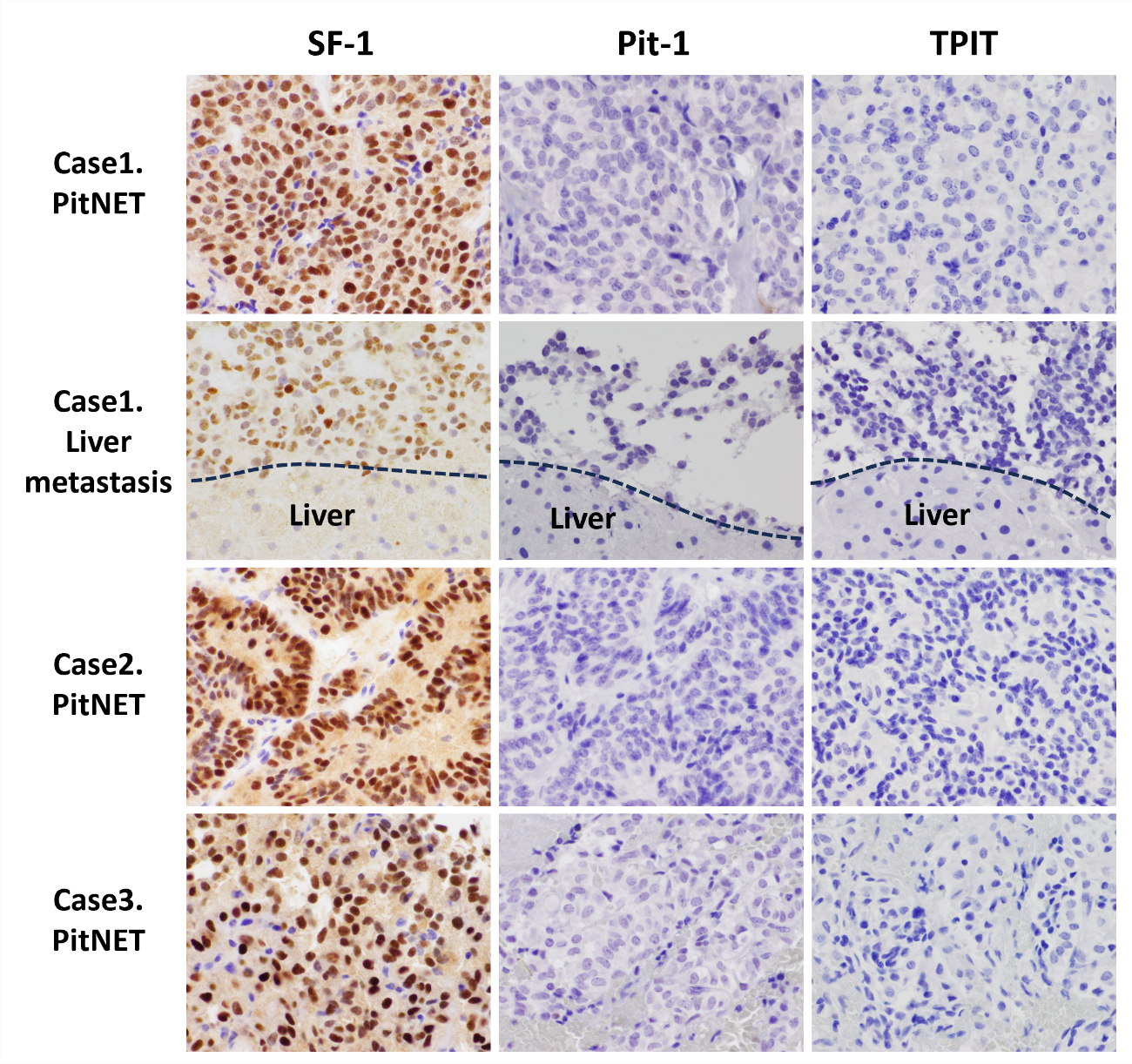


**Supplemental Figure 4: Immunohistochemistry for pituitary lineage transcription factors in Cases 1–3**

Representative images of steroidogenic factor-1 (SF-1/NR5A1), pituitary-specific transcription factor 1 (PIT1/POU1F1), and T-box pituitary transcription factor (TPIT/TBX19). Tumor cells show strong nuclear SF-1 positivity consistent with gonadotroph lineage, with negative staining for PIT1 and TPIT across cases. Hematoxylin counterstain. PitNET: pituitary neuroendocrine tumor.

**
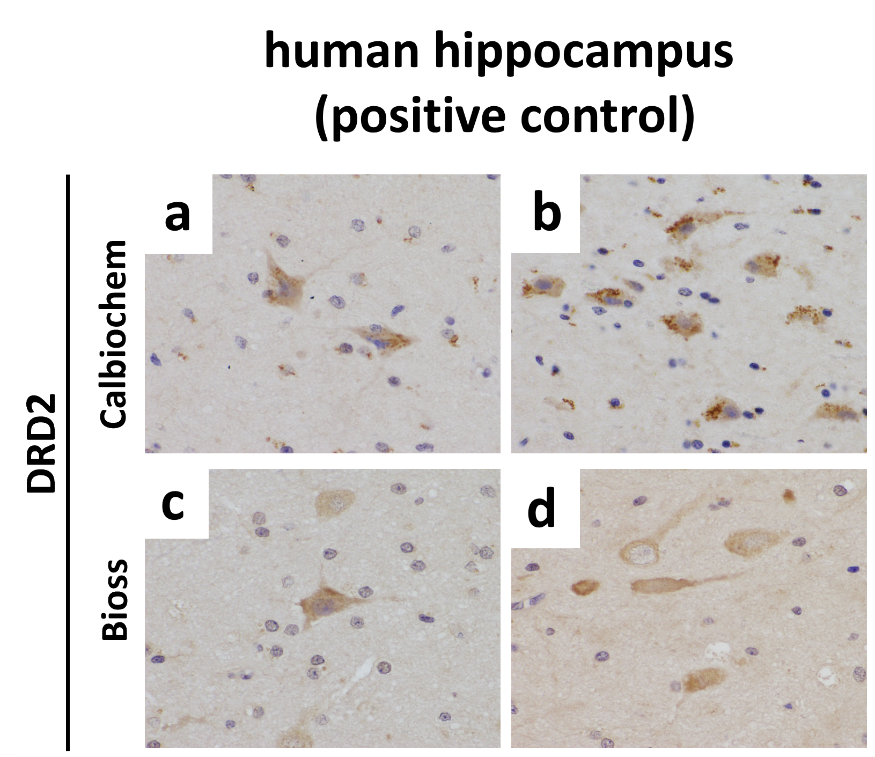
**

**Supplemental Figure 5. Positive-control immunohistochemistry for dopamine receptor D2 (DRD2) in human hippocampus.**

(a,b) Calbiochem (cat. no. 324393, Darmstadt, Germany), dilution 1:500 and (c,d) Bioss (cat. no. bs-1008R, Woburn, MA, USA), dilution 1:200; human hippocampal tissue with known D2-positive cells was used as the positive control for the antibodies. The Calbiochem antibody occasionally showed stronger cytoplasmic and focal nuclear labeling, with granular, intensely staining foci; however, overall cytoplasmic staining patterns were largely concordant between the two antibodies. Hematoxylin counterstain.
